# Engineering Non-Heme Mono- and Dioxygenases for Biocatalysis

**DOI:** 10.5936/csbj.201209011

**Published:** 2012-10-23

**Authors:** Adi Dror, Ayelet Fishman

**Affiliations:** a Department of Biotechnology and Food Engineering, Technion-Israel Institute of Technology, Haifa, 32000, Israel

## Abstract

Oxygenases are ubiquitous enzymes that catalyze the introduction of one or two oxygen atoms to unreactive chemical compounds. They require reduction equivalents from NADH or NADPH and comprise metal ions, metal ion complexes, or coenzymes in their active site. Thus, for industrial purposes, oxygenases are most commonly employed using whole cell catalysis, to alleviate the need for co-factor regeneration. Biotechnological applications include bioremediation, chiral synthesis, biosensors, fine chemicals, biofuels, pharmaceuticals, food ingredients and polymers. Controlling activity and selectivity of oxygenases is therefore of great importance and of growing interest to the scientific community. This review focuses on protein engineering of non-heme monooxygenases and dioxygenases for generating improved or novel functionalities. Rational mutagenesis based on x-ray structures and sequence alignment, as well as random methods such as directed evolution, have been utilized. It is concluded that knowledge-based protein engineering accompanied with targeted libraries, is most efficient for the design and tuning of biocatalysts towards novel substrates and enhanced catalytic activity while minimizing the screening efforts.

## Introduction

Biocatalysis involves the utilization of biological substances, enzymes or whole-cell systems, for the synthesis of valuable organic molecules in processes traditionally based on the use of chemical synthesis [1, 2]. In recent years, the use of biocatalysis in industrial processes has become more common as it offers efficient tools for transformation of natural or synthetic materials under mild reaction conditions, reduced energy and raw material consumption. Furthermore, biocatalysis enables the synthesis of new chemicals and decreases the generation of waste and toxic side-products, as compared to the equivalent chemical catalyst [3–6].

One group of biocatalysts with special interest for the chemical industry is the oxygenases. Oxygenases are enzymes that catalyze the introduction of one (monooxygenases) or two (dioxygenases) oxygen atoms into an organic substance. They typically use molecular oxygen (O_2_) as the oxygen donor and most of them require reduction equivalents usually supplied from NADH or NADPH by electron transfer proteins [7–10]. Most oxygenases are cofactor-dependent enzymes requiring metal-ions (e.g., copper or iron), metal-ion complexes (e.g., heme, Fe-S cluster) or coenzymes (e.g., flavin, pterin, pyrroloquinoline quinone) [7–10].

Oxygenases are attractive biocatalysts as they react with a vast variety of organic molecules and enable improved product purity by highly chemo-, regio- and stereo-selectivity in contrast to the poor selectivity of the strong oxidizing agents and the extreme reaction conditions of traditional organic chemical synthesis [7–9]. Various industrial applications based on oxygenases already exist. In the textile industry lacasses have been used for indigo dye bleaching in denim finishing [10, 11] using commercial enzymes such as DeniLite™ (Novozyme, Novo Nordisk, Denmark) and Zylite (Zytex Pvt. Ltd., Mumbai, India). A whole cell hydroxylation process of heteroarenes such as pyridine was developed by Lonza using *Pseudomonas putida* ATCC 33015 cells combining several oxidation steps by intrinsic mono- and dioxygenases [12–14]. This process was used for the production of 5-methylpyrazine-2-carboxylic acid, an intermediate for the production of an antilipolytic drug, by whole cell oxidation of 2,5-dimethylpyrazine [13].

Oxygenases hold a great potential for new applications in many fields, such as: textile, food, environment (biodegradation and bioremediation), biosensors, organic synthesis (chiral and asymmetric synthesis, polymers, pharmaceuticals), and biofuels [10, 15–18]. However, their potential is still far from being fulfilled. The practical use of oxygenases compared with other industrial enzymes such as hydrolases, lyases and isomerases, is limited by many factors [7, 8, 18, 19]. Many oxygenases have poor stability in unnatural environments as most of them are membrane-associated multicomponent complexes. In addition, their function depends on reduction equivalents (e.g., NADH or NADPH) that need regeneration by electron transfer proteins [7, 18, 19]. The cofactor regeneration is one of the main limitations of industrial biooxidation processes as the oxidation of every substrate molecule requires one molecule of NAD(P)H. The supplementation of the latter compounds in large amounts will make the production costs economically unfavorable [9]. Cofactor regeneration has been addressed by different approaches such as chemical, electrochemical, photochemical and enzymatic means. These possibilities were recently reviewed by Torres et al. [9]. A whole-cell system is an alternative solution to overcome some of these problems. However, in this case some disadvantages include generation of by-products by enzymes present in the host, low oxygen mass transfer, toxicity of substrates and products, limited substrate uptake and insufficient catalytic rate [18, 19]. In order to overcome the limitations discussed above, some modifications should be made to fit the process conditions to the available oxygenase properties (bioprocess engineering), or on the other hand the biocatalyst should be improved to make it suitable for chemical manufacturing process conditions (e.g., pH, temperature, organic solvents and pressure) and demands (e.g., substrate specificity and range, high catalytic rate and production yields) [4, 6, 18].

The promising commercial potential of oxygenases, especially their high selectivity, provided a motivation for many protein engineering studies in the past two decades. Efforts were made to modify the structure and catalytic properties of oxygenases to suit industrial applications. In recent years, advances in molecular biology techniques, bioinformatics, computational methods and screening methods expanded the protein engineering “tool box” and opened new possibilities to design improved biocatalysts in terms of stability, catalytic activity, substrate scope and selectivity. This article reviews selected studies regarding non-heme oxygenases from recent years (2007-2012), which present diverse protein engineering approaches for targeting the desired biocatalyst property. A summary of these protein engineering reports is presented in Table 1.

**Table 1:** Selected protein engineering studies of non-heme oxygenases

Enzyme/source	Mutagenesis method	New variant^a^	Improved property	Ref.
StyMO/ *P. putida* CA-3	Random mutagenesis by 3 rounds of epPCR. Expressed in *E. coli* BL21 (DE3).	K245E/A420T, A179T/K426N, R87C/V303I	8-12–fold improved oxidation rate of styrene → (*S*)-styrene oxide and indene → (*1S, 2R*)-indene oxide (ee 97%)	[45]

StyMO/ P*seudomonas sp*. LQ26	Site directed mutagenesis based on X-ray crystal structure and molecular docking. Expressed in *E. coli* BL21 (DE3).	R43A, L44A, N46A	Improved epoxidation of α-methyl-styrene without changing the ennantioselectivity.	
		L44A, L45A, N46A	1.5-, 2-, and 3.3-fold improved epoxidation of α-ethyl-styrene without change in ennantioselectivity	[46]

T4MO/ *P. mendocina* KR1		I100A/D285I	52-fold improved hydroxylation of PEA → *m*-Tyr (91%) and *p*-Tyr (7%). 12.6- fold improved sulfoxidation of methyl-*p*-tolyl sulfide → (S)-methyl-*p*-tolyl sulfoxide (80% ee pro S).	
		I100G/D285 I	14.1-fold improved sulfoxidation of methyl-*p*-tolyl sulfide → (S)-methyl-*p*-tolyl sulfoxide (82% ee pro S).	[36]
	Site-specific saturation mutagenesis (using NNN codon). Expressed in *E. coli* TG1.	I100A/D285Q	85-fold improved hydroxylation of PEA → *m*-Tyr (93%) and *p*-Tyr (7%).	
		D285S	1.7-fold improved oxidation of styrene → styrene oxide without change in enantioselectivity.	
		I100S, I100G and I100A	34-, 35- and 36-fold improved hydroxylation of *p*-Tyr → HTyr (65%, 48% and 98% conversion) respectively. Di-hydroxylation of PEA → HTyr.	[37]
	Random mutagenesis by epPCR. Expressed in *E. coli* TG1.	S395C	15-fold improved activity. Novel hydroxylation of o-Tyr → 2,3-dihydroxyphenyl ethanol	
	Site-specific saturation mutagenesis (using NNN codon). Expressed in *E. coli* TG1.	I100G	1.7-fold fold improved oxidation rate of methyl phenyl sulfide to the corresponding sulfoxide with increased enantiomeric excess to 98% (pro-*S*). 11-fold improved oxidation rate of methyl p-tolyl sulfide and altered selectivity from 41% pro-R to 77% pro-S.	[33]
	Site directed mutagenesis based on statistical model predictions. Expressed in *E. coli* TG1.	I100A/E214G/D285Q	190-fold higher initial oxidation rate of PEA. 2.6-fold higher initial oxidation rate of toluene (the natural substrate)	[41]

TOM/ *Burkholderia cepacia* G4	Site-specific saturation mutagenesis (using NNN codon). Expressed in *E.coli* TG1.	V106A, V106S and V106E	25-, 28- and 39-fold improved hydroxylation of PEA with change in regiospecificity.	[37]
		V106M	2-fold improved oxidation rate of methyl phenyl sulfide to the corresponding sulfoxide with increased enantiomeric excess (pro-*S*) to 88%.	[33]

ToMO/ *Pseudomonas sp*. strain OX1	Site directed mutagenesis based on computational model. Expressed in E. coli strain JM109.	F176I, F176L and F176T	Improved regioselectivity in PEA hydroxylation → *p*-tyrosol (98%).	[38]
		E103G/F176T, E103G/F176I	Improved regioselectivity in PEA hydroxylation → *p*-tyrosol (98%) and increased *k*_*cat*_ value.	

sMMO/ *M. trichosporium* strain OB3b	Site directed mutagenesis based on crystal structure. Expressed in M. trichosporium strain SMDM.	L110G, L110C	Novel oxidation of toluene → *m*-cresol and biphenyl → 3-hydroxybiphenyl. Inverted regioselectivity in oxidation of ethyl benzene →3-ethylphenol and 4-ethylphenol. Novel oxidation of ethyl benzene →2-ethylphenol.	[60]
		L110Y, L110R	Inverted regioselectivity in naphthalene oxidation (74.8 and 70.6% 1-naphtol respectively). Inverted regioselectivity in oxidation of ethyl benzene →3-ethylphenol and 4-ethylphenol. Novel oxidation of ethyl benzene →2-ethylphenol.	

PAMO/ *Thermobifida fusca*	Site-specific saturation mutagenesis (using position specific codon degeneracy) based on sequence alignment with homologues.	S441A/A442W/L443Y/S444T	High R-enantioseletivity (E = 70)	[54]
		Different sequence combinations at positions 441–444.	Different enantioselectivity as a result of the different combinations.	
	Site-specific saturation mutagenesis (using NNK codon) based on bioinformatics approach combining sequence alignment, and docking model.	P440F	160-fold improved rate of the kinetic resolution of 2-phenylcyclohexanone. Expanded substrate scope and high enantioselectivity.	
		P440L, P440I, P440Y, P440W, P440N, P440H	Expanded substrate scope of 2-substituted cyclohexanone and high enantioselectivity.	[51]
	Site-specific saturation mutagenesis (using NDT codon) based on docking model and crystal structure.	Q93N/P94N	Expanded substrate scope of 2-substituted cyclohexanone with high enantioselectivity.	[48]

AtdA/ *Acinetobacter sp*. strain YAA	Site-specific saturation mutagenesis (using the NNS codon) based on homology model. Expressed in *E. coli* strain JM109.	V205A	Expanded substrate scope.	[61]
		I248L	1.7- fold improved conversion rate of aniline and 2.1-fold fold improved conversion rate of 2,4-dimethylaniline	
	Site-specific saturation mutagenesis followed by one round of random mutagenesis (epPCR). Expressed in E. coli strain JM109.	V205A/I248L/S404C	8.9-, 98.0-, and 2.0-fold improved activity on aniline, 2,4-dimethylaniline and 2-isopropylaniline respectively, compare to V205A. 3.5-fold improved activity on 2,4-dimethylaniline vs. wild type.	[62]

BPDO/ *Burkholderia xenovorans* LB400	Family shuffling of soil DNA.	Q179E/T237M/I247M/Q255H/I258V/A268S/Y277F/L285M	Change in the regiospecificity. Oxydize 2,2-dichlorobiphenyl on carbons 5 and 6.	[50]

BPDO/*P. pseudoalcaligenes* strain KF707	Hybrid construction followed by site-specific saturation mutagenesis (using the NNS codon).	T324A/I325L, T324L/I325I	7-hydroxyflavone → 2-(2,3-dihydroxyphenyl)-7-hydroxy-chromen-4-one and 5,7-dihydroxyflavone (chrysin) → 2-(2,3-dihydroxyphenyl)-5,7-dihydroxy-chromen-4-one. trans-chalcone → 3-(2,3-dihydroxyphenyl)-1-phenylpropan-1-one and further into 1,3-bis-(2,3-dihydroxyphenyl)-propan-1-one.	[49]

IsoB/ *A. radioresistens* S13	Site-specific saturation mutagenesis (using NNK codon) and site directed mutagenesis based on homology modeling. Expressed in *E. coli BL21 (DE3)*	L69A	Inversion of specificity on catechol to 4-chloro-catechol.	
		A72S, A72G	*k*_*cat*_ enhancement towards chlorinated substrates.	[63]

C23O/ *Pseudomonas sp*. CGMCC2953	Site directed mutagenesis based on homologue X-ray structure and Modip- web-based disulfide bond prediction server. Expressed in E. coli BL21 (DE3).	A229C/H294C	Improved thermostability and tolerance to alkaline environment.	[64]

AkbA*/ Rhodococcus sp*. strain DK17	Site-directed mutagenesis. Expressed in *E. coliBL21 (DE3)*.	L266F	Improved hydroxylation of biphenyl to 2-hydroxybiphenyl and 3-hydroxybiphenyl. Improved hydroxylation of *o*-xylene to 3, 4-dimethylphenol.	[65]

NBDO/ *Comamonas sp*. strain JS765	Saturation mutagenesis (using NNK codon) based on X-ray structure, previous work and HotSpot Wizard. Expressed in *E. coli* BL21 (DE3).	V207I	Improved pro-*R* enantioselectivity and decreased activity.	
		V207A	Reversed pro-*S* enantioselectivity and decreased activity.	
		N258A	Improved enantioselectivity (pro-*R*) and 1.6- 2.0- 2.8- and 3.5-fold improved oxidation of thioanisole, *p-*tolyl, cl-thioanisole and br-thioanisole respectively.	[66]
		F293H	2.4- 4.8- 7.4- and 17-fold improved oxidation of thioanisole, *p-*tolyl, cl-thioanisole and br-thioanisole, respectively.	
	Site-directed mutagenesis.	N258A/F293H	Improved enantioselectivity (pro-*R*) and 1.7- 4.6- 7.14- and 26.7-fold improved oxidation of thioanisole, *p-*tolyl, cl-thioanisole and br-thioanisole, respectively.	

LadA/ *G.thermodenitrificans* NG80-2	Random mutagenesis by epPCR, followed by site-specific saturation mutagenesis (using NNK codon). Expressed in *E. coli* BL21 (DE3).	A102D, A102E, L320V, L320A, F146C/N376I, F146Q/N376I, F146E/N376I, F146R/N376I, F146N/N376I	2–3.4-fold improved hydroxylation of hexadecane.	[67]

a New enzyme represented by the amino acid substitution (in a single letter code), slashes indicate that all substitutions are in the same enzyme. Abbreviations: StyMO - styrene monooxygenase, T4MO - toluene-4-monooxygenase, Tyr- tyrosol, PEA – 2-phenylethanol, BPDO - biphenyl dioxygenase, HTyr – hydroxytyrosol, sMMO - soluble methane monooxygenase, PAMO - phenylacetone monooxygenase, AtdA - aniline dioxygenase, IsoB - catechol 1,2-dioxygenase, C23O - catechol-2,3-dioxygenase, AkbA - *o*-xylene dioxygenase, NBDO - nitrobenzene dioxygenase, LadA - alkane monooxygenase.

## Protein engineering

**Figure 1:**
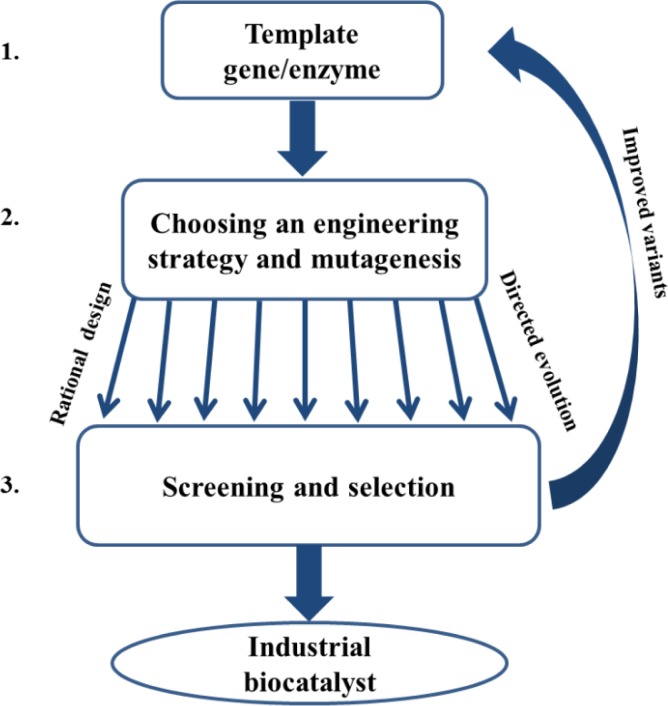
A schematic diagram illustrating the protein engineering process. **1.** Choosing the gene template encoding for the desired enzyme and cloning into a suitable host. **2.** Choosing the engineering strategy and mutagenesis methods that will give the best results in the shortest time using the minimum effort. **3.** Screening or selection for variants possessing the desired property. The best variants can be used as the template for further rounds of mutagenesis and screening for the same property or a different one. The protein engineering process continues until the biocatalyst with the desired properties is obtained.

Protein engineering refers to the alteration of an existing protein structure in order to improve desired traits for targeted applications [20, 21]. It is generally composed of three major steps: 1) Choosing the template based on the desired application and the available enzyme; 2) Choosing the engineering strategy and mutagenesis approach. The decision depends on the data available (e.g. crystal structure, homology modeling, sequence alignment with homologues, and empirical results) and existing screening or selection methods. The mutagenesis strategies range between rational design (performed by site specific or saturation mutagenesis) to random directed evolution (performed by error prone PCR or family shuffling), and often the final strategy is a combination of the two; 3) Screening or selection for discovering those variants possessing the improved desired properties [20, 21]. The best variants of the mutagenesis cycle are isolated and characterized and in most cases serve as the template for further rounds of mutagenesis in order to improve the specific property or to add a different one (e.g., improve the catalytic rate and then improve the stability). A schematic diagram illustrating the protein engineering process is presented in Figure 1.

The most popular and studied strategies in protein engineering are “rational design” and “directed evolution”. Both strategies have been used separately or in tandem to tailor enzymes for specific applications [1, 20, 21]. Rational protein design was originally based mostly on the available structural data for rationally choosing positions in the protein sequence for alteration to different amino acids. Nowadays, the data can be obtained not only from 3-D x-ray crystal structures, but also from homology modeling, docking models, sequence alignment of the protein and its homologues, previous experimental results, and statistical models available in the internet. The main limitation of the rational approach is that our understanding of the correlation between protein structure and function is limited, and the attempt to predict the influence of amino acid substitutions in specific positions may end in adverse results. As opposed to rational protein design, directed evolution of enzymes is based on the Darwinian principle of mutation and selection and not on a detailed understanding of enzyme structure and function [2, 22]. In this approach a diversity is generated by random mutagenesis (e.g., error prone PCR - epPCR) [23], or gene recombination (e.g., family shuffling) [24] for the creation of a mutant gene library which covers a large sequence space. The library is screened for the desired property and the improved variants are isolated for the next round of mutagenesis and screening. The main drawback of this approach is the need for a high throughput screening or selection method which may not be available. The screening problem is emphasized when the desired property is regio- or enantioselectiviy as in many cases those reactions products can be analyzed mainly by low throughput screening methods (e.g., GC, HPLC) [25].

In recent years, the main approach in protein engineering studies is data-driven protein design. This tactic combines both strategies in order to create relatively small size “smarter” libraries (10^2^-10^4^) by utilizing structural knowledge (crystal structure, homology models, empirical results) and statistical methods to predict beneficial alterations for the desired trait (e.g., thermostability, selectivity) [6, 20]. This approach is supported by the increasing amount of data in databases (more than 75,000 protein structures are available in the protein data bank), next-generation DNA sequencing, bioinformatics, and computational tools. Creation of smaller libraries reduces the screening efforts and enables screening assays closer to the industrial application conditions. For example, a protein engineering approach such as iterative saturation mutagenesis (ISM) [26], combines selection of few positions in the enzyme which may influence the desired property using structural data, and evolutionary randomization by stepwise applying saturation mutagenesis at every position. The best variant of every library is used as the template for saturation mutagenesis at a different position [25, 26]. This approach was combined with saturation mutagenesis using reduced amino acid alphabets such as NNK codon degeneracy (32 codons/20 aa) or NDT (12 codons/12 aa) codon degeneracy ( N represents A, T, G or C, and K represents G or T, D represents A, G or T) for the creation of relatively smaller libraries with high functionality [25]. Many other tools and techniques for the generation of industrial biocatalysts are available and reviewed by Bommarius et al. [20] and by Bornscheuer et al. [27]. A good protein engineering process is the one that will give the desired results in the shortest period of time with a reasonable effort. However, the choice of a good protein engineering strategy depends on one's “tools box”, information intensity (crystal structure, previous studies and experimental results), mutagenesis methods, and screening/selection techniques [21].

## Engineering non-heme oxygenases

### Toluene/xylene/styrene monooxygenases

Selective hydroxylation of aromatic compounds is of great interest to organic chemists. Members of the toluene monoxygenase (TMOs) family were extensively investigated for their ability to perform highly regio- and enantio-selective hydroxylation of such compounds [28–31]. TMOs belong to a group of four component, non-heme, diiron alkene/aromatic monooxygenases [32]. Their hydroxylase is composed of two subunits in (αβγ)_2_ quaternary structure and the electron transfer from the NADH reductase to the hydroxylase is mediated by a Rieske-type [2Fe-2S] ferredoxin [32]. In recent years members of this monooxygenase family were the focus of protein engineering studies aiming to improve their catalytic ability in terms of rate, substrate scope and selectivity.

**Figure 2:**
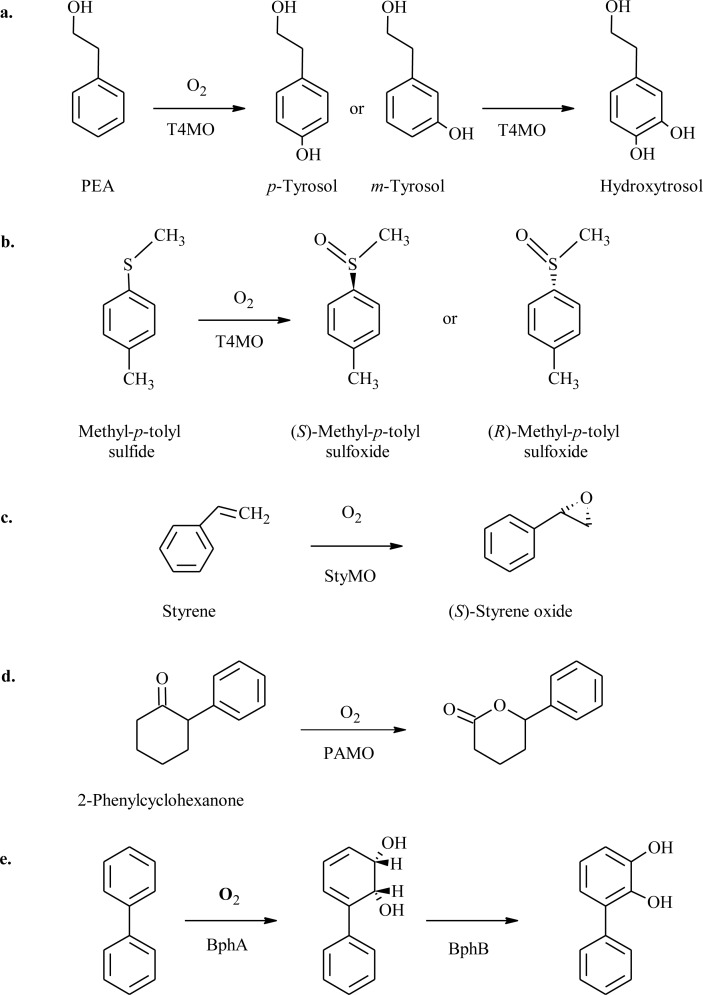
Selected non heme mono- and dioxygenase reactions: **a.** PEA hydroxylation by toluene monooxygenases (TMOs) to *p*- and *m*-tyrosol and successive hydroxylation to form hydroxytyrosol (HTyr) [36–38, 41]. **b.** Methyl-*p*-tolyl sulfide oxidation by T4MO to (*S*)-, (*R*)-methyl-*p*-tolyl sulfoxide [33, 36]. **c.** Styrene epoxidation by StyMO to (*S*)-styrene oxide [45]. **d.** 2-Phenylcyclohexanone oxidation by phenyl acetone monooxygenase (PAMO) [48]. **e.** Biphenyl hydroxylation to 2,3-dihydroxybiphenyl by BPDO (BphA – biphenyl dioxygenase, BphB – dihydrodiol dehydrogenase) [49, 50].

Toluene 4-monooxygenase (T4MO) of *Pseudomonas mendocina* KR1 and toluene *ortho*-monooxygenase (TOM) of *Burkholderia cepacia* G4 were investigated for their ability to perform enantioselective oxidation reactions of aromatic sulfides [33]. Position V106 in the α-hydroxylase subunit of TOM and the analogous position, I100 of T4MO, were chosen for mutagenesis based on previous work by Wood and co-workers in which this residue was found to influence the enzyme regiospecificity in hydroxylation reactions [34, 35]. Saturation mutagenesis libraries, expressed in *E. coli* and tested in whole-cell systems, were screened for improved activity and enantioselectivity on methyl phenyl sulfide and methyl *para*-tolyl sulfide using gas chromatography (GC) (Figure 2b). TOM variant V106M showed an improved oxidation rate of 3.0 nmol/min/mg protein for methyl phenyl sulfide to the corresponding sulfoxide compared with 1.6 for the wild-type enzyme, together with an increased enantiomeric excess of 88% (pro-*S*) vs. 51% for the wild type. T4MO variant I100G showed an increased oxidation rate by 1.7-fold with an improved enantiomeric excess of 98% (pro-*S*). In addition, I100G oxidized methyl *para*-tolyl sulfide at a higher rate of 11-fold compared to the wild type and changed the selectivity from 41% pro-*R* to 77% pro-*S* [33].

These results highlighted the importance of residues V106 (TOM) and its analogue I100 (T4MO) for tuning activity and selectivity of unnatural substrates. It was hypothesized that TOM V106 possess an important role in the proper positioning of the substrate with respect to the diiron atoms. Based on the experimental results it was suggested that a decreased oxidation rate and impaired enantioselectivity were a result of a substrate not properly aligned in the active site [33].

**Figure 3:**
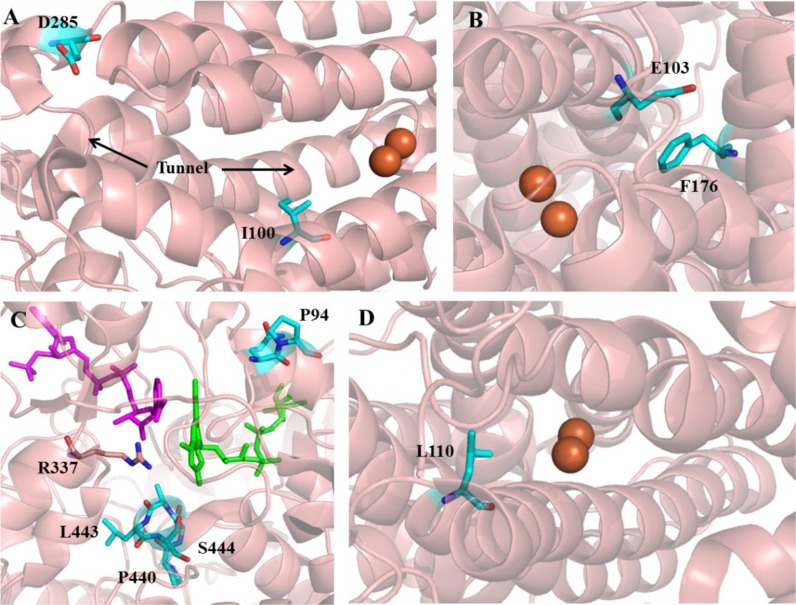
**A.** The tunnel and active site region of the α-subunit of T4MO hydroxylase (PDB code: 3DHG [56]). Key residues which were subjected to mutagenesis in the tunnel entrance (D285) and in the active site (I100) are marked in cyan. The Fe atoms are colored as brown spheres. The helix segment between residues I61-Y82 was truncated to enable the visualization of the tunnel and the active site. **B.** The active site of the α-subunit of ToMO hydroxylase (PDB code: 1T0Q [57]). Key residues which were subjected to mutagenesis are colored by cyan (E103 and F176). The Fe atoms are colored as brown spheres. The helix segment between residues E486-Q491 was truncated to enable the visualization of the active site pocket. **C.** The active site region of PAMO with bound FAD (green) and NADP^+^ (dark pink) (PDB code: 2YLR [58]). Key residues which were subjected to mutagenesis are colored by cyan (P440, S441, A442, L443, S444, Q93 and P94). Active site residue R337 is colored by light pink. **D.** The active site region of the sMMO hydroxylase (PDB code: 1XU3 [59]). Residue L110 which was subjected to mutagenesis is colored by cyan. The Fe atoms are colored as brown spheres. All structures were visualized using PyMOL.

Further investigation was done by Brouk et al. in order to have a better understanding on the influence of residues in the tunnel leading to the active site vs. active site residues of T4MO [36]. The chosen positions for saturation mutagenesis were residue D285 which is located at the tunnel entrance distant from the active site, and residue I100 located in the active site close to the diiron center (Figure 3A) [33]. Using three different substrates, the libraries were screened for their activity, regioselective hydroxylation of 2-phenylethanol (PEA) and enantioselective oxidation of styrene and of methyl *p*-tolyl sulfide (Figure 2a-b). This study revealed that mutations at position 285 at the tunnel entrance improved the oxidation rate without affecting the regio- and enantioselectivity. The best amino acid substitution for enhanced activity was found to be substrate dependent. D285I and D285Q enhanced the oxidation rate of PEA and methyl *p*-tolyl sulfide (large and bulky substrates) by 8–11-fold compared to wild type, while variant D285S improved the oxidation rate of styrene to styrene oxide by 1.7-fold. The activity and selectivity of T4MO towards all three substrates was influenced by substitutions at position I100. An additive or even synergistic effect on the enzyme activity and selectivity was further achieved when site directed mutagenesis was applied to combine the best substitutions of both positions. I100A/D285Q and I100A/D285I enhanced the oxidation rate of PEA by 85- and 52-fold respectively, with improved regiospeceficity towards *m*-tyrosol (91-93%, respectively) [36].

Based on the results it was concluded that residue 285 which is located in the entrance to the tunnel leading to the active site influences the substrate/product flow to/from the active site without changing the regio- and enantioselectivity. In contrast, residue 100 which is part of the hydrophobic gate in the active site entrance affected both activity and selectivity, by improving flux into the diiron center and by changing the size of the active site pocket [36, 37].

Toluene *o*-xylene monooxygenase (ToMO) from *Pseudomonas* sp. strain OX1was also investigated for the ability to hydroxylate PEA to tyrosol (Figure 2a). Notomista et al. [38] used a computational strategy based on Monte Carlo approach to predict the effect of amino acid substitutions in the active site of the ToMO hydroxylase subunit on the enzyme's regioselectivity on aromatic substrates [39]. A kinetic model of the active site with productive and unproductive enzyme-substrate complexes was built in order to locate residues and possible substitutions that will improve the hydroxylation activity of ToMO on PEA and tune the regioselectivity from *m*-tyrosol (90% in the wild type) to *p*- tyrosol. Based on the model, positions E103 and F176 were predicted to influence the regioselectivity and catalytic efficiency of the reaction as both of them contribute to the active site cavity edge (Figure 3B). Site directed mutagenesis was used to create variant E103G and three different variants of F176 (I, L, T) and six double mutants of E103G/F176 (I, L, T, A, S, V). All F176 variants hydroxylated PEA exclusively to *p*-tyrosol. E103G showed similar regioselectivity as wild type, but the double mutated variants showed improved catalytic efficiency and regioselectivity (Table 1) [38].

TMOs were engineered to perform successive double hydroxylation of PEA to form hydroxytyrosol (HTyr), a valuable natural phenolic compound with exceptional antioxidant, antimicrobial and anticarcinogenic activities (Figure 2a) [37]. Saturation mutagenesis libraries of TOM V106 and T4MO I100 and a random mutagenesis library of T4MO at the α- and γ-hydroxylase subunits, generated by epPCR, were screened on PEA, *o*-, *m*- and *p*-tyrosol. The screening method was based on the instability of the catechol derivatives which auto-oxidize and polymerize forming red-orange dye [40]. Colonies showing a red halo were chosen for whole cell biotransformation analysis with product measurement performed with GC/MS. Both targeted and random libraries generated mutants with improved oxidation activity compare to wild type. However, T4MO mutants I100A, I100S and I100G and TOM mutants V106S, V106A and V106E performed the di-hydroxylation of PEA to form the desirable HTyr. In addition, T4MO S395C variant from the random mutagenesis library showed a 15-fold improved activity on PEA and hydroxylated *o*-tyrosol to form 2,3-dihydroxyphenyl ethanol, a reaction not performed by wild-type. Position 395 is distant from the active site but was found to influence both the activity and selectivity of the enzyme [37]. These results emphasize the strength of classical directed evolution methods in targeting important positions influencing the enzyme activity which are not expected to be found by rational design methods.

Further improvement of T4MO hydroxylation activity on PEA to form HTyr was achieved by integrating statistical modeling into protein engineering [41]. The statistical model of Nov and Wein [42], which addresses the protein design problem by capturing characteristics of protein structure-activity relationship, was used to predict 16 beneficial mutant combinations (out of ∼13,000 possibilities) in two rounds of statistical analysis. Seven double or triple mutants were suggested based on an initial data set from previous studies [36, 37]. These mutants showed a 4.6-fold improvement in the average activity compare to the average activity of the initial data set variants. A second round of statistical analysis generated nine mutants with 3-5 point mutations. The second round mutants had 7.3-fold improvement in the average activity compared to the initial data set. T4MO variant I100A/E214G/D285Q was the best enzyme with an initial PEA oxidation rate of 4.4 ± 0.3 nmol/min/mg protein which is190-fold higher than wild type and also 2.6-fold higher than the wild type activity on toluene, the natural substrate [41]. This study demonstrated that the combination of statistical modeling with rational design and directed evolution, may lead to a highly active biocatalyst in minimum time and screening efforts.

New crystal structures of the T4MO hydroxylase subunit (T4moH) bound to 4-bromophenol, and the complex of T4moH with the effector protein T4moHD bound to the natural product *p*-cresol have been reported recently by Bailey and coworkers [43]. These structures can be used for further engineering of T4MO to enhance its activity in desired reactions.

Another enzyme which was extensively studied is styrene monooxygenase (StyMO). StyMO is a flavoenzyme consisting of two components, StyA – an FAD dependent monoxygenase, and StyB – an NADH reductase. StyMO catalyzes predominantly the (*S*)-epoxidation of *m*- and *p*-styrene derivatives to form the corresponding styrene oxides (Figure 2c) [4, 44]. *Pseudomonas putida* CA-3 StyMO genes, *StyAB*, were subjected to three rounds of random mutagenesis by epPCR in order to improve the rate formation of styrene oxide and indene oxide [45]. The screening method was based on a colorimetric method of indole biotransformation to indigo. The best clones were isolated and used for the biotransformation of the styrene and indene to the corresponding oxides. Although the screening method was based on a different substrate than the target, 9-12-fold improvement was obtained for styrene and indene oxidation while the enantioselectivity remained high (Table 1). Most of the mutations of the improved variants were located in *StyA* together with an insertion mutation that changed the translation reading frame and added 19 amino acids to the StyA subunit [45].

Rational design was used to improve the activity of StyMO from *Pseudomonas sp*. LQ26 towards α-ethylstyrene [46]. Four mutants (R43A, L44A, L45A, and N46A) were designed based on a docking model [47] using the x-ray crystal structure of the StyMO epoxidase subunit from *P. putida* S12 (PDB code 3IHM). All variants displayed different substrate preference. The most active variant was L45A with 3-fold improved activity on α-ethylstyrene [46].

## Baeyer-Villiger monooxygenases

Baeyer-Villiger monooxygenases (BVMO) are flavin-dependent monooxygenases which catalyze the Baeyer-Villiger oxidation of ketones to form lactones or esters. The use of BVMO to catalyze these reactions enantioselectively is an attractive alternative to the traditional metalo- organo-catalysts used by organic chemistry [51, 52].

Phenyl acetone monooxygenase (PAMO) from *Thermobifida fusca*, the only known thermostable BVMO [53], was the subject of protein engineering studies conducted by the Reetz group [48, 51, 54]. Three different strategies were used to expand the narrow substrate range of PAMO. Positions 441-444 on a loop having a direct contact with the binding pocket were chosen for mutagenesis based on previous studies [55] and on sequence alignment with seven other BVMOs (Figure 3C). A degenerate codon was used to introduce all possible amino acids occurring in a specific position according to the sequence alignment [54]. Only 1700 clones were screened for the oxidative resolution of 2-phenylcyclohexanone (Figure 2d) in a whole-cell system with 145 variants exhibiting improved activity compare to the wild type. Some of the best variants were isolated and tested for their activity and thermostability. These variants displayed different enantioselectivity (pro-*S* or pro-*R*) and had high thermostability as the WT did. The highest enantioselectivity (*E*-Value = 70, pro-*R*) was displayed by variant S441A/A442W/L443Y/S444T [54]. According to the PAMO x-ray structure, residues 441–444 are located near R337 of the active site (Figure 3C) which has been suggested to stabilize the Criegee intermediate [54]. The results confirmed that mutagenesis of these residues can influence the substrate acceptance and enantioselectivity [54].This study also demonstrates the successful use of degenerate codon alphabet in several targeted positions, for reducing the screening efforts while achieving great improvement in catalytic activity.

Bioinformatics was used to further improve these results. Position 440 was chosen for mutagenesis based on a docking model of the ligand phenylacetone in wild type PAMO (PDB code 1W4X) (Figure 3C) [51]. A library was generated by saturation mutagenesis using NNK codon degeneracy and screened for the ability to perform oxidative kinetic resolution of 2-ethylcyclohexanone. The activity test was done with isolated enzymes in conjunction with an alcohol dehydrogenase from *Thermoanaerobacter ethanolicus* for NADPH regeneration using isopropanol as the reductant [51]. Seven variants with improved activity were found (Table 1). The best variants were further evaluated on other 2-substituted cyclohexanone substrates. All variants attained an expanded substrate scope of 2-substituted cyclohexanones and high enantioselectivity while maintaining their thermostability. The most active variant, P440F, had 160-fold improved rate for the kinetic resolution of 2-phenylcyclohexanone compare to the wild type [51]. P440 is located on a flexible loop in the second shell surrounding the active site (Figure 3C). Mutagenesis of a proline residue most likely changed the loop configuration which resulted in changes in substrate acceptance, enantioselectivity, and oxidation rate [51].

A different approach to influence the catalytic activity and increase the substrate range of PAMO, was the use of mutagenesis in a distant region from the active site with the aim to trigger allosterically-induced domain movements and to create a new binding pocket [48]. Based on the PAMO crystal structure, positions Q93/P94 which are located in the N-terminal region of an α-helix were chosen for mutagenesis (Figure 3C). Saturation mutagenesis using the NDT codon degeneracy was performed simultaneously on both positions and activity was measured in a cell free system on the model reaction of oxidative kinetic resolution of 2-ethylcyclohexanone [51]. Q93N/P94N was the most active variant found from screening 400 transformants. Its activity was further tested on 2-substituted cyclohexanones demonstrating vast substrate acceptance by the mutant with high enantioselectivity. However, when mutagenesis was performed separately at each position, no active enzymes were found [51].

## Soluble methane monooxygenases

Soluble methane monooxygenase (sMMO) is a catalytically versatile enzyme that can catalyze the oxygenation of unreactive methane to methanol, as well as a wide range of other substrates such as, naphthalene, biphenyl, carbon monoxide and ammonia [60, 68]. sMMO is a multicomponent enzyme comprising of an (αβγ)_2_ hydroxylase component, an NAD(P)H-dependent reductase with an FAD and an Fe_2_S_2_ center, and an effector protein, all encoded by a multigene operon *mmoXYBZDC* [60, 68]. Its catalytic abilities present great potential for the development of a robust biocatalyst for synthetic chemistry and bioremediation [60, 68]. However, the main obstacle concerning protein engineering of sMMO is the difficulty to achieve functional expression of this multicomponent enzyme in *E. coli* [68]. A partial solution addressing this problem was developed by Smith and coworkers, in which mutagenesis was performed on the relevant gene in a specially designed vector in *E. coli* cells. The mutant gene was then transferred using a shuttle plasmid by conjugation to *Methylosinus trichosporium* with a partially deleted *mmoXYBZDC* operon [60, 68, 69]. This expression system was used for site directed mutagenesis of sMMO from *M. trichosporium* strain OB3b at position L110 (Figure 3D) [60]. Based on the hydroxylase crystal structure and on comparison to homologues, L110 located in the entrance to the active site was suspected to influence the substrate acceptance for large aromatic molecules and the regioselectivity of the enzyme [60]. Indeed, all mutants (L110G, L110C, L110R and L110Y) showed a change in their regioselectivity compare to the wild type (Table 1). Variants L110R and L110Y had an inverted regioselectivity in naphthalene hydroxylation (1-naphtol, 70.6 and 74.8% respectively). Novel products were generated by all variants in the hydroxylation of toluene (*m*-cresol) and ethyl benzene (2-ethylphenol) with moderate regioselectivity, and L110G and L110C hydroxylated biphenyl to 3-hydroxybiphenyl. However, the mutants were not able to hydroxylate larger aromatic hydrocarbons such as anthracene and phenanthrene [60]. Based on the research results it was suggested that residue 110 has a crucial role in coordinating the substrate in the active site pocket and controlling the precision of regioselectivity, rather than limiting the size acceptance of substrates to the active site [60]. These results underscore the great potential of this monooxygenase as a biocatalyst; nevertheless, a suitable expression system still limits the practical applications.

## Dioxygenases

Dioxygenases are non-heme Rieske type NADPH-dependent enzymes which catalyze the introduction of two oxygen atoms into an organic substrate. This heterogeneous group has an important role in the natural biodegradation and industrial bioremediation of aromatic compounds, such as polychlorinated biphenyls (PCBs), naphthalane and polycyclic arenes [7, 8, 70]. Dioxygenases are also multicomponent enzyme complexes composed of an oxygenase subunit, an iron-sulfur flavo-protein reductase, and a ferredoxin [7]. In recent years, protein engineering has been used to improve the catalytic rate, regioselectivity and thermostability of dioxygenases for biosynthesis and bioremediation purposes.

Biphenyl dioxygenase (BPDO) catalyzes the first step in biphenyl biodegradation (Figure 2e). The regiospecificity of BPDO from *Burkholderia xenovorans* LB400 was successfully changed to accept 2,2’-dichlorobiphenyl by the use of family shuffling of *bphA* genes from polychlorinated biphenyl-contaminated soil DNA combined with site specific mutagenesis [50]. BPDO oxidation of the refractory flavonoids, 7-hydroxyflavone and 5,7-dihydroxyflavone (Chrysin) to their vicinal diol forms was enhanced by constructing a hybrid of the dioxygenase large α subunit, BphA1, followed by saturation mutagenesis using NNK codon at positions 324 and 325 of *bphA1*. The hybrid was composed of amino acid sequence between positions 268–397 of BphA1 from *P. pseudoalcaligenes* strain KF707 which was exchanged with the corresponding sequence of *P. putida* strain KF715. The hybrid *bphA1* was cloned together with *bphA2A3A4BC* genes from strain KF707 in *E. coli* JM109 for expression and whole cell biotransformation [49].

Site directed mutagenesis based on previous studies [71] and molecular modeling of *o*-xylene dioxygenase (AkbA) from *Rhodococcus* sp. strain DK17 was used to improve its hydroxylation ability of biphenyl. Variant L266F had an elevated hydroxylation activity on biphenyl to produce 2-hydroxybiphenyl (2.43 vs. 0.1 mg/L) and 3-hydroxybiphenyl (1.97 vs. 0.03 mg/L) compare to wild-type [65].

Site-specific saturation mutagenesis based on homology modeling was used to extend the substrate range of aniline dioxygenase (AtdA) from *Acinetobacter* sp. strain YAA [61]. The best variant, V205A, was used as the template for another round of saturation mutagenesis in the active site residue I248 followed by one round of random mutagenesis to enhance the bioremediation activity of aromatic amines. Triple mutant V205A/I248L/S404C showed the most significant improvement as its activity was increased by 3.5-fold over the wild type on the carcinogenic aromatic amine, 2,4-dimethylaniline [62].

Nitrobenzene dioxygenase (NBDO) form *Comamonas sp*. strain JS765 was evaluated for the ability to perform enantioselective oxidation of aromatic sulfides to the corresponding sulfoxides. By examining different *para*-substituted alkyl aryl substrates, NBDO oxidation activity was found to be dependent on the substrate size [66]. In order to improve the oxidation activity and enhance the enantioselectivity, five positions, V207, F222, N258, F293 and N295, were chosen for saturation mutagenesis based on previous work [72, 73], alignment with homologue aniline dioxygenase [62] and the HotSpot Wizard [74]. HotSpot Wizard is a web server designed for the identification of residues favorable for mutagenesis in the enzyme structure (“hot spots”) [74]. The libraries were tested in a whole-cell biotransformation for the oxidation activity and enantioselectivity of four substrates, thioanisole, *p-*tolyl sulfide, chloro-thioanisole and bromo-thioanisole. Variant N258A which showed improved enantioselectivity (pro-*R*) and increased activity towards all substrates and variant F293H which showed enhanced activity and moderate enantioselectivity, were combined by site specific directed mutagenesis. The double mutant, N258A/F293H had improved enantioselectivity as variant N258A and improved activity towards all four substrates, especially towards thioanisole (26.7-fold improved oxidation) (Table 1) [66]. Variants V207I and V207A had decreased activity but with opposite enantioselectivity; V207I had improved pro-*R* enantioselectivity and V207A had the reversed pro-*S* enantioselectivity (Table 1) [66]. A substrate docking model was generated using AutoDock Vina [75] in order to obtain better understanding of the correlations between the substrate structure and the enzyme activity. Based on the model, it was suggested that the enantioselectivity and activity of NBDO is determined by the substrate positioning in the active site which is coordinated by hydrophobic interactions and steric considerations [66].

Catechols and chlorinated catechols are considered as carcinogenic and teratogenic pollutants which therefore must be removed from the environment [63]. Catechol dioxygenases and chlorocatechol dioxygenases play an important role in the detoxification of these pollutants. Site specific mutagenesis and site specific saturation mutagenesis based on homology modeling and sequence alignment were applied on catechol 1,2-dioxygenase (IsoB) from *A. radioresistens* S13 to fine-tune the catalytic properties and improve the activity on bulky catechol derivatives [63]. Variants at position L69 had inverted specificity favoring 4-chlorocatechol and enhanced activity on other chlorinated catechols while variants at position A72 enhanced the activity rate on chlorinated catechols [63].

Stability under industrial conditions is a major issue for the application of biocatalysts in a production process. Thus, the thermostability of catechol-2,3-dioxygenase (C23O) from *Pseudomonas* sp. CGMCC2953 was improved by introducing disulfide bonds [64]. Potential sites for forming disulfide bonds were analyzed using Modip, a web-based disulfide bond prediction server [76] and the chosen mutations were introduced by site specific mutagenesis. By introducing cysteine residues at positions A229 and H294 a disulfide bond was created. The mutagenesis outcome was indeed improved thermostability and enhanced alkali stability [64].

## Conclusions

Non-heme oxygenases are attractive biocatalysts for organic chemistry due to their high chemo-, regio-, and enantioselectivity. Protein engineering was applied to these enzymes mainly to enhance the catalytic rate, expand the substrate range for unnatural substrates and fine tune the selectivity. Properties affecting enzyme stability under reaction conditions, such as thermostability, were generally not studied. Many of the studies reviewed, used a data-driven protein engineering approach to successfully evolve new enzymes with improved catalytic properties. However, the classic strategy of directed evolution by random mutagenesis was proven to be an important tool as well, as it helped in locating positions distant from the active site affecting the enzyme catalytic activity, which are less likely to be found in a more rational approach.

An appropriate engineering strategy is one that allows reaching the target in the shortest time with minimum screening efforts. Directed evolution generated by random mutagenesis covers a large sequence space however requires high throughput screening of large libraries. For example, directed evolution by epPCR was used to enhance T4MO hydroxylation activity. By screening more than 3000 clones one mutant with 15-fold improved activity and altered selectivity was found [37]. Similarly, epPCR was applied to enhance the hexadecane hydroxylation activity of LadA. By screening 7,500 clones of a random mutagenesis library, 3 mutants with 2-fold improved activity were found. The improved variants were further subjected to site specific saturation mutagenesis which resulted in a mutant performing 3.4-fold better than the wild type [67]. The data-driven approach on the other hand, focuses on the mutagenesis of targeted positions with high potential to influence the desired property along with the use of restricted codon degeneracy for the creation of relatively small libraries. This strategy was successfully used to improve PAMO activity by generating two site specific saturation mutagenesis libraries of 200 clones each. The best mutant of this study, exhibited 160-fold improvement in rate for the kinetic resolution of 2-phenylcyclohexanone [51]. This strategy was also applied to improve the activity of T4MO hydroxylation of phenylethanol to hydroxytyrosol. By generating two site specific saturation mutagenesis libraries of 300 clones each, and combining the best mutations of each library, an 85-fold improvement was achieved [36, 37]. Further improvement was obtained by using statistical model predictions for the best mutation combination. By generating only 16 variants out of ∼13,000 possibilities, a triple variant with 190-fold improved activity was obtained [41]. In both studies successful results were obtained while minimizing the screening efforts to only few hundreds compare to the large libraries which are inherent in directed evolution protocols (10^3^-10^6^) [1, 2]. With the ever-increasing generation of new data on enzymes, whether by new crystal structures or sequences, or through reports by numerous laboratories involved in enzyme engineering, we believe that the data-driven approach will become more popular and widespread.

Another observation from the studies reviewed, was that most of the researchers employed whole cell systems for the biotransformation to circumvent the need for co-factor regeneration. The issue of NAD(P)H regeneration still requires perfection in order to fulfill the maximum potential of oxygenases as industrial biocatalysts.
